# *Drosophila* Signal Peptidase Complex Member Spase12 Is Required for Development and Cell Differentiation

**DOI:** 10.1371/journal.pone.0060908

**Published:** 2013-04-03

**Authors:** Erin Haase Gilbert, Su-Jin Kwak, Rui Chen, Graeme Mardon

**Affiliations:** 1 Department of Molecular and Human Genetics, Baylor College of Medicine, Houston, Texas, United States of America; 2 Department of Pathology and Immunology, Baylor College of Medicine, Houston, Texas, United States of America; 3 Program in Developmental Biology, Baylor College of Medicine, Houston, Texas, United States of America; 4 Department of Neuroscience, Baylor College of Medicine, Houston, Texas, United States of America; 5 Department of Ophthalmology, Baylor College of Medicine, Houston, Texas, United States of America; 6 Human Genome Sequencing Center, Baylor College of Medicine, Houston, Texas, United States of America; University of Massachusetts Medical School, United States of America

## Abstract

It is estimated that half of all proteins expressed in eukaryotic cells are transferred across or into at least one cellular membrane to reach their functional location. Protein translocation into the endoplasmic reticulum (ER) is critical to the subsequent localization of secretory and transmembrane proteins. A vital component of the translocation machinery is the signal peptidase complex (SPC) - which is conserved from yeast to mammals – and functions to cleave the signal peptide sequence (SP) of secretory and membrane proteins entering the ER. Failure to cleave the SP, due to mutations that abolish the cleavage site or reduce SPC function, leads to the accumulation of uncleaved proteins in the ER that cannot be properly localized resulting in a wide range of defects depending on the protein(s) affected. Despite the obvious importance of the SPC, *in vivo* studies investigating its function in a multicellular organism have not been reported. The *Drosophila* SPC comprises four proteins: Spase18/21, Spase22/23, Spase25 and Spase12. Spc1p, the *S. cerevisiae* homolog of Spase12, is not required for SPC function or viability; *Drosophila spase12* null alleles, however, are embryonic lethal. The data presented herein show that *spase12* LOF clones disrupt development of all tissues tested including the eye, wing, leg, and antenna. In the eye, *spase12* LOF clones result in a disorganized eye, defective cell differentiation, ectopic interommatidial bristles, and variations in support cell size, shape, number, and distribution. In addition, *spase12* mosaic tissue is susceptible to melanotic mass formation suggesting that *spase12* LOF activates immune response pathways. Together these data demonstrate that *spase12* is an essential gene in *Drosophila* where it functions to mediate cell differentiation and development. This work represents the first reported *in vivo* analysis of a SPC component in a multicellular organism.

## Introduction

Processing by the signal peptidase complex (SPC) is critical to the localization and function of secretory and membrane proteins which must enter the endoplasmic reticulum (ER) before they can be directed to their final destination. As proteins are transferred into the ER, the SPC cleaves the signal peptide sequence (SP), an N-terminal stretch of amino acids – usually 20–30 residues in length – that directs proteins to the ER [1]. SPs possess a tripartite structure that includes a positively charged amino terminal domain, a 7–13 residue hydrophobic domain, and a hydrophilic domain that includes the cleavage site [2]. While the SP sequence is not conserved, the properties associated with each domain are static and ensure that SP-bearing polypeptides are recognized by the cell and translocated into the ER [1], [3].

Signal peptidases have been extensively studied in yeast and bacteria, yet little has been done to investigate their role in multicellular organisms. In *S. cerevisiae*, four proteins, Sec11, Spc1p, Spc2p and Spc3p, comprise the SPC [4], [5] (Table 1). Sec11 and Spc3p are required for SPC catalytic function and cell viability. Temperature-sensitive *sec11* and *spc3p* mutants accumulate uncleaved SPC targets at non-permissive temperatures, indicating that both are required for SP cleavage [6]–[8]. Spc1p and Spc2p do not have catalytic function and are dispensable for SPC cleavage activity and viability at normal growth temperatures. However, over-expression of Spc1p attenuates the *sec11* temperature-sensitive phenotype [5], while depletion of Spc2p at high temperatures leads to the accumulation of uncleaved protein [9], suggesting that Spc1p and Spc2p contribute to SPC function in yeast although the mechanism has yet to be identified.

**Table 1:** The SPC is conserved from yeast to humans.

Yeast	*Drosophila*	Human	*Drosophila*-Human
			Identity(Similarity)
Sec11	Spase18/21	SPC18 and SPC21	70(91)
Spc1p	Spase12	SPC12	39(75)
Spc2p	Spase25	SPC25	33(65)
Spc3p	Spase22/23	SPC22/23	33(65)

*S. cerevisiae* and *Drosophila* SPC both contain four proteins, while the human complex is comprised of five members.

In mammals, the SPC consists of five subunits: SPC18, SPC21, SPC22/23, SPC12 and SPC25 [10]. SPC18 and SPC21 have high identity to each other [11] and are homologous to Sec11 [6], [12], [13]. SPC22/23 is homologous to Spc3p [14]–[17] while SPC12 and SPC25 are homologous to Spc1p and Spc2p, respectively [5], [9], [18], [19] (Table 1). SPC18, SPC21 and SPC22/23 are single-pass transmembrane proteins, the bulk of which reside within the ER lumen. SPC12 and SPC25 are double-pass transmembrane proteins each containing a small lumenal domain, while the N- and C-termini of both are cytosolic [11]. SPC18, SPC21, and SPC22/23 have catalytic function and the residues required for cleavage activity are localized to the ER lumen [11].

Four SPC homologs have been identified in *Drosophila*: Spase18/21, Spase22/23, Spase12 and Spase25 (Table 1). Spase18/21 is homologous to yeast Sec11, as well as mammalian SPC18 and SPC21 [20]. ER vesicles (microsomes) purified from *Drosophila* embryos and added to an *in vitro* translation system results in cleavage of murine myeloma light-chain IgG, demonstrating that the *Drosophila* SPC is functionally conserved [21].

Despite playing a key role in protein sorting, *in vivo* studies of SPC function in metazoans have not been reported. We have used the *Drosophila* eye as a model system to investigate the role of *spase12* and the SPC in a higher eukaryote. The eye originates from a developmental structure called the eye imaginal disc, an epithelial monolayer of cells that begin to differentiate during the third instar larval stage. The eye continues to develop through larval and pupal stages into a highly organized array comprised of approximately 800 unit eyes (ommatidia). Each ommatidium contains eight photoreceptor cells and four cone cells enclosed by two primary, six secondary, and three tertiary pigment cells, as well as three interommatidial bristles (IOBs) [22], [23]. The genetic approaches available in *Drosophila*, coupled with the well characterized development and structure of the eye, make it an ideal model for developmental studies.

In this report, we characterize *spase12* loss-of-function (LOF) phenotypes in the *Drosophila* eye through clonal analysis. Our findings show that *spase12* mutants are embryonic lethal, while *spase12* LOF clones result in developmental defects in all tissues tested. Specifically, *spase12* LOF in the *Drosophila* eye leads to errors in cell differentiation. Together, these data indicate that *spase12* is required for viability, development, and differentiation.

## Results

### *spase12* is required for development

**Figure 1:**
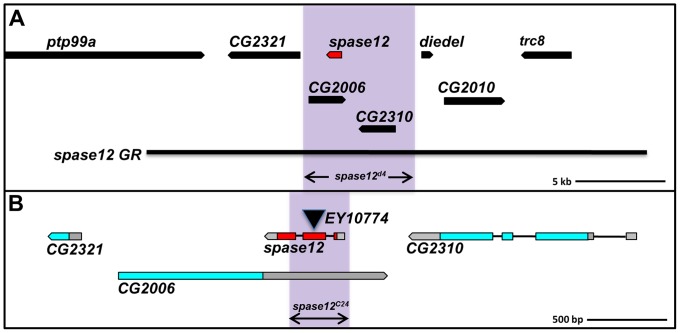
*spase12* mutant alleles. (A) *spase12^d4^* is a 4 kb deletion (purple shaded region). (B) *spase12^EY10774^* contains a transposon inserted in the second exon. (B) *spase12^C24^* is a 303 bp deletion (purple shaded region). *spase12 GR* (A) is a 29 kb genomic construct.

To determine the effects of *spase12* LOF on *Drosophila* development, we utilized three *spase12* mutant alleles. s*pase12^d4^* is a lethal 4 kb deletion that removes two additional genes of unknown function: *CG2006* and *CG2310* (Figure 1A). *spase12^EY10774^* carries a P-element inserted into the second exon of *spase12* (Figure 1B). *spase12^C24^* is a 303 bp deletion generated through imprecise excision of *EY10774* that removes the first and second exons of *spase12*, as well as a portion of exon three (Figure 1B). These three alleles are embryonic lethal, recessive, and fail to complement one another.

**Figure 2:**
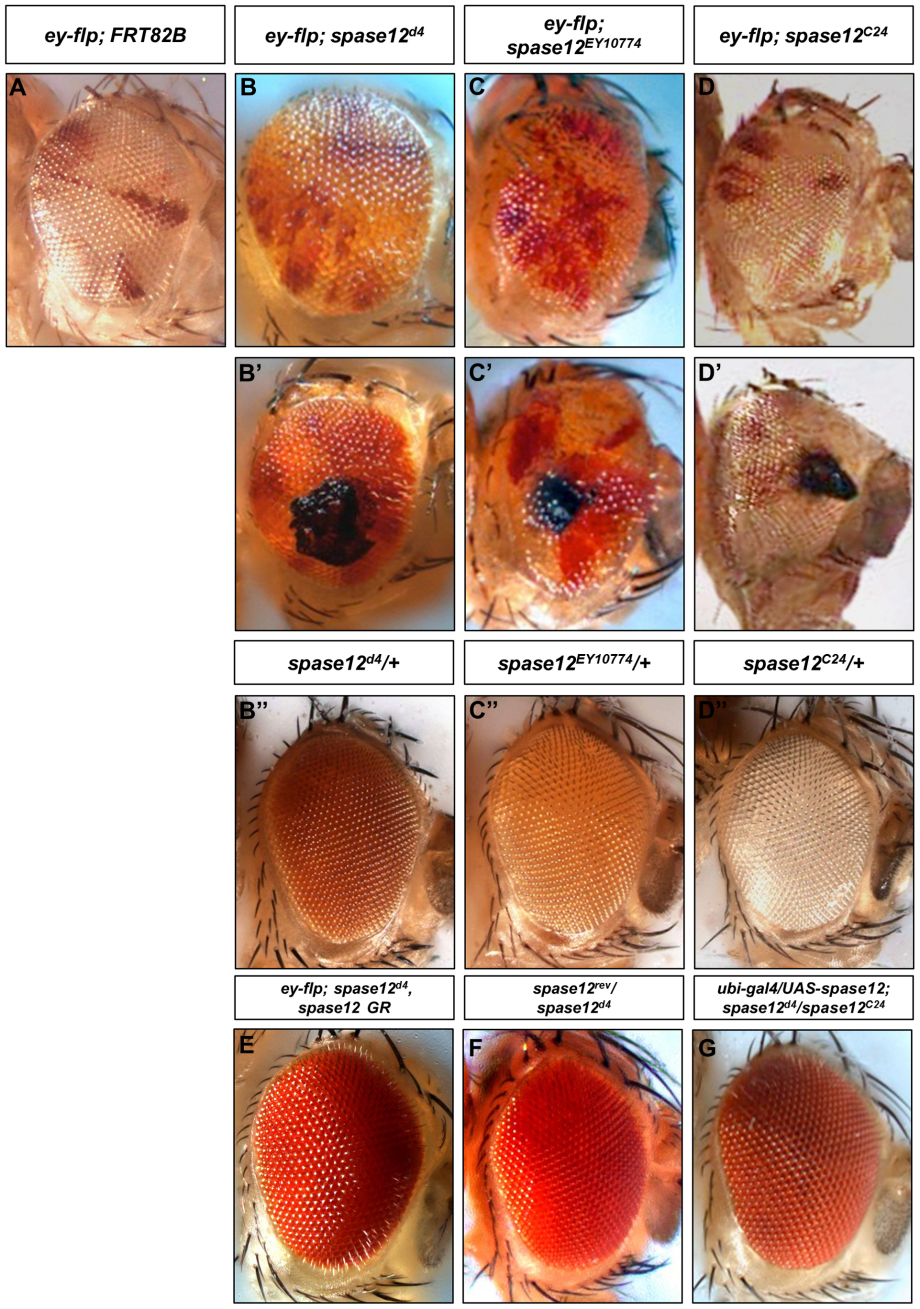
*spase12* LOF results in a disorganized eye, loss of pigmentation, and melanotic mass formation. (A) *yw ey-flp/+; FRT 82B P[w+] cl/FRT 82B* where *w+* marks control tissue and *w-* marks the clone. *yw ey-flp/+; FRT 82B P[w+] cl/FRT 82B spase12^d4^ P[w+] (ey-flp; spase12^d4^)* (B), *yw ey-flp/+; FRT 82B P[w+] cl/FRT 82B spase12^EY10774^ P[w+] (ey-flp; spase12^EY10774^)* (C) and *yw ey-flp/+; FRT 82B P[w+] cl/FRT 82B spase12^C24^ (ey-flp; spase12^C24^)* (D) eyes are disrupted compared to the control (A). Clonal tissue in *ey-flp; spase12^d4^* eyes (B) appears lighter in color than *spase12^d4^/+* which exhibits a strong *P[w+]* eye color (B''). *spase12^EY10774^/+* (C''). *spase12^C24^/+* (D''). Examples of melanotic masses in *ey-flp*; *spase12^d4^* (B'), *spase12^EY10774^* (C'), and *spase12^C24^* (D') mosaic eyes. A single copy of *spase12 GR* rescues *spase12^d4^* in *yw ey-flp/+; FRT 82B P[w+] cl/FRT 82B spase12^d4^ P[w+], spase12 GR P[w+]* (E). *spase12^rev^* complements *spase12^d4^* (F). Ubiquitous expression of *UAS-spase12* rescues *spase12* phenotypes in *w; ubi-gal4/UAS-spase12; spase12^d4^ P[w+]/FRT 82B spase12^C24^* flies (G).

To investigate *spase12* function, we focused on the eye, which is unnecessary for viability and the development of which is well characterized. Using the *ey-flp* cell lethal (cl) method, clones were induced in the eye imaginal disc during the second larval instar stage and that comprise approximately 90% of the adult eye field [24]. *yw ey-flp/+; FRT 82B P[w+] cl/FRT 82B spase12^d4^ P[w+] (ey-flp; spase12^d4^), yw ey-flp/+; FRT 82B P[w+] cl/FRT 82B spase12^EY10774^ P[w+]* (*ey-flp; spase12^C24^*), and *yw ey-flp/+; FRT 82B P[w+] cl/FRT 82B spase12^C24^ (spase12^C24^)* animals have a disorganized adult eye (Figure 2B–D). Additionally, loss of pigmentation is observed in *spase12^d4^* clones where clonal tissue appears to be a light yellow-orange color (Figure 2B) rather than the strong red *P[w+]* color observed in *spase12^d4^*/+ heterozygotes (Figure 2B''). This specific phenotype cannot be observed with *spase12^EY10774^* (weak *P[w+]* allele with light orange eye color) or *spase12^C24^* (*w-* allele) clones (Figure 2C'', D'').

We tested whether *spase12* LOF is responsible for the observed phenotypes using rescue with both genomic DNA constructs and the Gal4/UAS system. A single copy of a 29 kb *spase12* genomic rescue *(spase12 GR)* construct – which extends 10 kb both 5′ and 3′ of the *spase12* locus and is intended to cover the deleted region and encompass all necessary regulatory elements (Figure 1A), rescues all known *spase12^d4^* eye phenotypes (Figure 2E). *spase12^rev^*, a precise excision of the *EY10774* insertion, complements *spase12^d4^* suggesting that phenotypes observed in *spase12^EY10774^* mutants are caused by the P-element insertion and *spase12* LOF (Figure 2F). *UAS-spase12* expressed under a ubiquitous promoter is sufficient to rescue *spase12^d4^/spase12^C24^* lethality and eye phenotypes (Figure 2G). Because *spase12* is embedded in the 3′ UTR of another gene, *CG2006* (Figure 1B), rescue with *UAS-spase12* further demonstrates that *spase12* is responsible for the observed phenotypes and that *CG2006* LOF does not significantly contribute to lethality or developmental defects.

**Figure 3:**
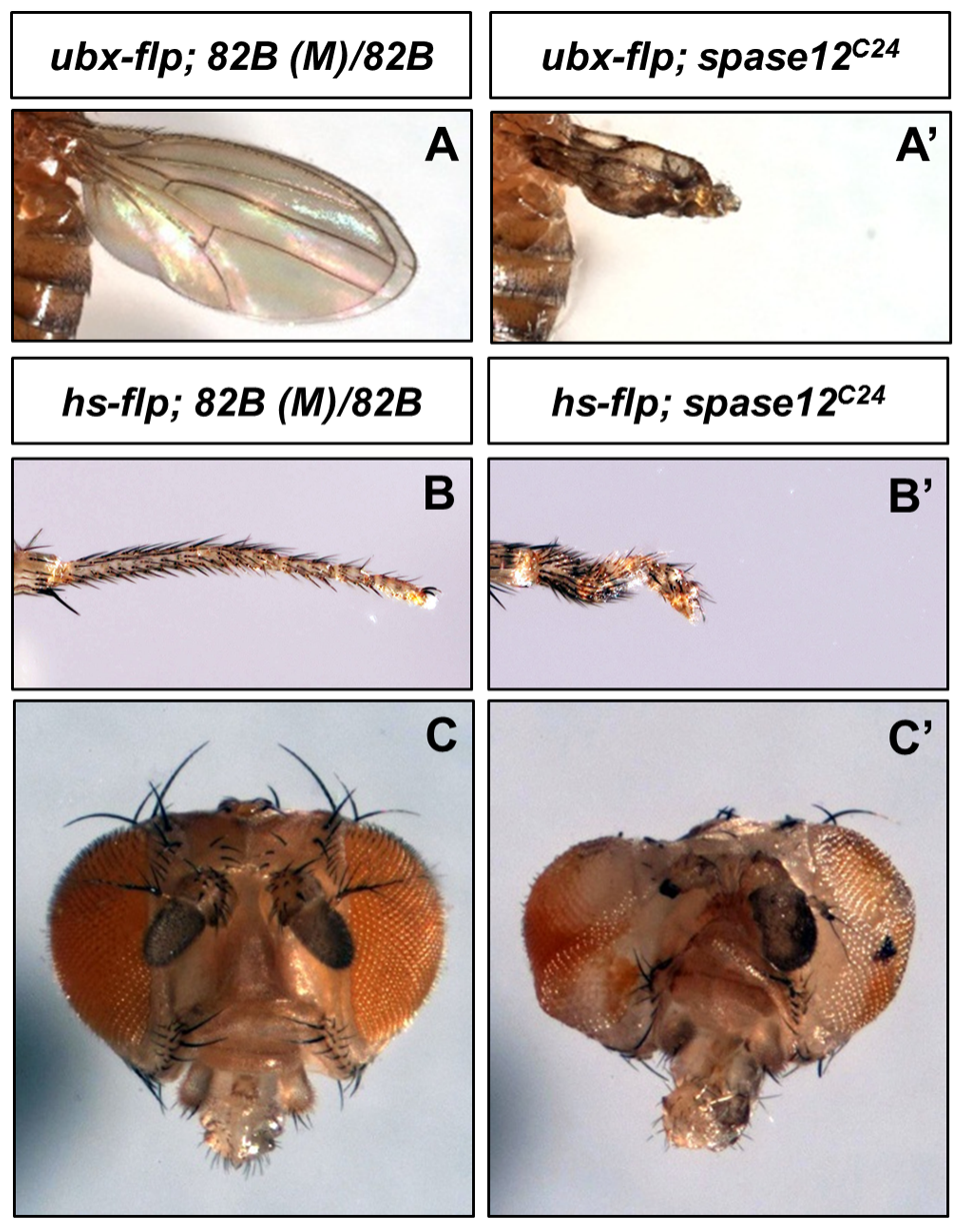
*spase12* LOF disrupts development in multiple tissues. *yw ubx-flp/+; FRT 82B (M) P[w+ ubi-GFP]/FRT* control (A), *yw ubx-flp/+; FRT 82B (M) P[w+ ubi-GFP]/FRT 82B spase12^C24^* wings are crumpled and melanized (A'). Clones in the distal portion of *yw hs-flp/+; FRT 82B (M) P[w+ ubi-GFP]/FRT 82B spase12^C24^* legs (B') are twisted and stunted compared to *yw hs-flp/+; FRT 82B (M) P[w+ ubi-GFP]/FRT 82B* control (B). *yw hs-flp/+; FRT 82B (M) P[w+ ubi-GFP]/FRT 82B* control (C). Clones in the head region of *yw hs-flp/+; FRT 82B (M) P[w+ ubi-GFP]/FRT 82B spase12^C24^* result in eye, bristle, and antennal defects as well as melanotic mass formation (C').

Beyond the eye phenotypes observed, *spase12* expression is also required for the development of other tissues. s*pase12^C24^* clones were generated in the wing using *ubx-flp Minute (M)* and result in crumpled, melanized wings (Figure 3A'). Clones induced via heat shock 48 hours after egg lay in *yw hs-flp/+; FRT 82B (M) P[w+ ubi-GFP]/FRT 82B spase12^C24^* larvae yields flies with developmental defects in multiple tissues including the leg, which may become twisted and stunted (Figure 3B'), and in the head region resulting in eye, bristle, and antennal defects as well as melanotic masses (Figure 3C').

### *spase12* mosaic eyes have disrupted retinal structure

**Figure 4:**
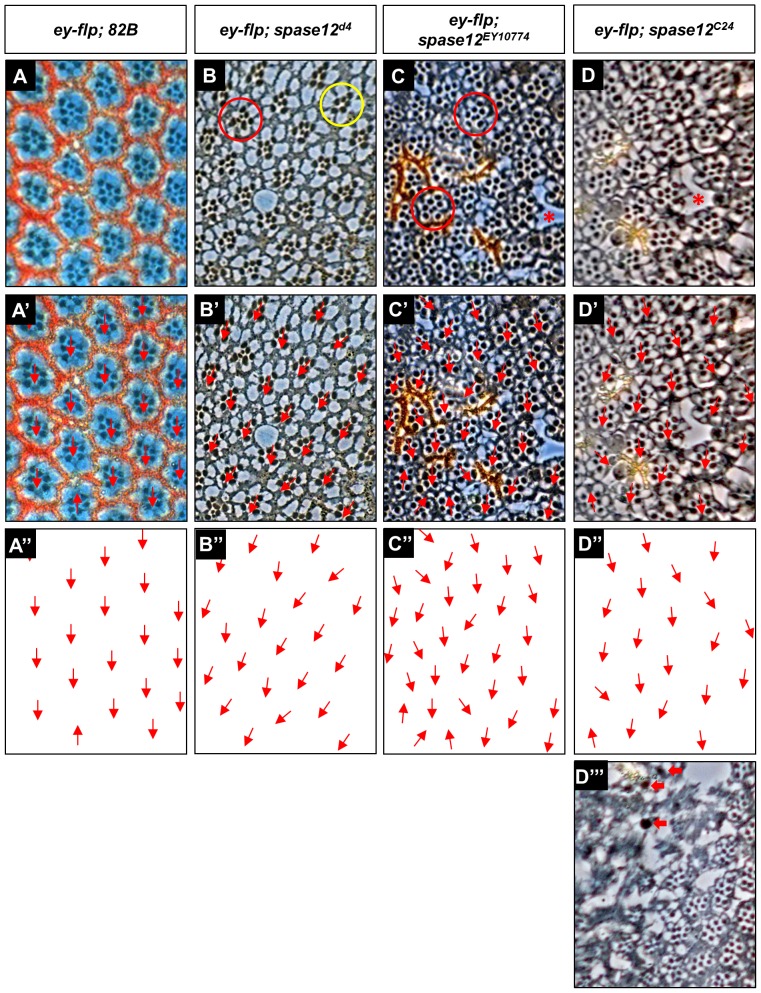
spase12 adult eyes have ectopic and missing rhabdomeres and polarity defects. Thin plastic sections of *yw ey-flp/+; FRT 82B P[w+] cl/FRT 82B* control (A), *yw ey-flp/+; FRT 82B P[w+] cl/FRT 82B spase12^d4^ P[w+] (ey-flp; spase12^d4^) *(B), *yw ey-flp/+; FRT 82B P[w+] cl/FRT 82B spase12^EY10774^ P[w+] (ey-flp; spase12^EY10774^) *(C) and *yw ey-flp/+; FRT 82B P[w+] cl/FRT 82B spase12^C24^ (ey-flp; spase12^C24^) *(D) mosaic eyes are disorganized and exhibit spacing defects between ommatidia (red asterisks) and varying rhabdomere numbers. Red circles mark ommatidia with an ectopic inner rhabdomere while yellow circles mark ommatidia with a missing inner rhabdomeres. Polarity of individual ommatidia within *ey-flp; 82B* (A', A''), *ey-flp; spase12^d4^*(B', B''), *ey-flp; spase12^EY10774^*(C', C''), and *ey-flp; spase12^C24^* (D', D'') is represented by red arrows. Section through *ey-flp; spase12^C24^* melanotic mass (D''') reveals degenerating tissue with large black dots consistent with dying cells (red arrows).

Adult eyes were sectioned to assay whether the disorganized appearance of *spase12* mosaic eyes is indicative of a compromised retinal structure. In sections of wild-type animals (Figure 4A), ommatidia are arranged in a stereotyped, lattice pattern and aligned such that rows of ommatidia share the same polarity (Figure 4A'–A''). However, sections of *ey-flp; spase12^d4^* (Figure 4B–B''), *ey-flp*; *spase12^EY10774^* (Figure 4C–C''), and *ey-flp; spase12^C24^* (Figure 4D–D'') animals reveal widely disorganized ommatidia with polarity defects. Furthermore, multiple ommatidia have varying numbers of rhabdomeres – the light-sensing organelles of photoreceptor cells. Within a single wild-type ommatidium, rhabdomeres appear as dark circles arranged in a trapezoidal pattern and indicate the presence of photoreceptors. Each wild-type ommatidium includes eight photoreceptors, but only seven rhabdomeres can be observed in each section because the inner R7 rhabdomere is positioned above the more basal R8 rhabdomere (Figure 4A). In *ey-flp; spase12* mutants, both ectopic and missing rhabdomeres are observed (Figure 4B, C). Variations in rhabdomere number suggest that there are incorrect numbers of photoreceptor cells in *ey-flp; spase12* mutant eyes. The appearance of supernumerary rhabdomeres could also be the result of a split rhabdomere or misplaced inner photoreceptor cells allowing for overlapping rhabdomeres. Additionally, spaces between ommatidia suggest possible retinal degeneration (Figure 4C, D).

### Loss of *spase12* causes melanotic mass formation and apoptosis

One of the most striking phenotypes observed in *spase12* mutants are black lesions called melanotic masses that occur in approximately 15% of *spase12* mosaic eyes (2B'–D'). *ey-flp*; *spase12* mutants typically eclose without lesions, which then appear within one to three days. They may appear and remain static or they may expand to cover the entire eye. *spase12* mosaic tissue in the wings (Figure 3A') and in the head region (Figure 3C') may also result in melanizations. Melanotic masses represent an inflammatory response that occurs when the immune system recognizes a foreign body or abnormal or dying tissue that is too large to be phagocytosed [25], [26]. Specialized hemocytes converge upon and encapsulate the threat forging an indestructible barrier that is subsequently melanized preventing further damage to the surrounding tissues [27], [28]. Melanizations in *ey-flp; spase12^C24^* animals are restricted to mosaic tissue suggesting that lesions are directly associated with abnormal tissue induced by *spase12* LOF rather than a defect in hemocyte function. Sections through melanotic masses in *ey-flp; spase12^C24^* animals (Figure 4D''') reveal degenerating tissue with large black inclusions that are consistent with dying cells.

**Figure 5:**
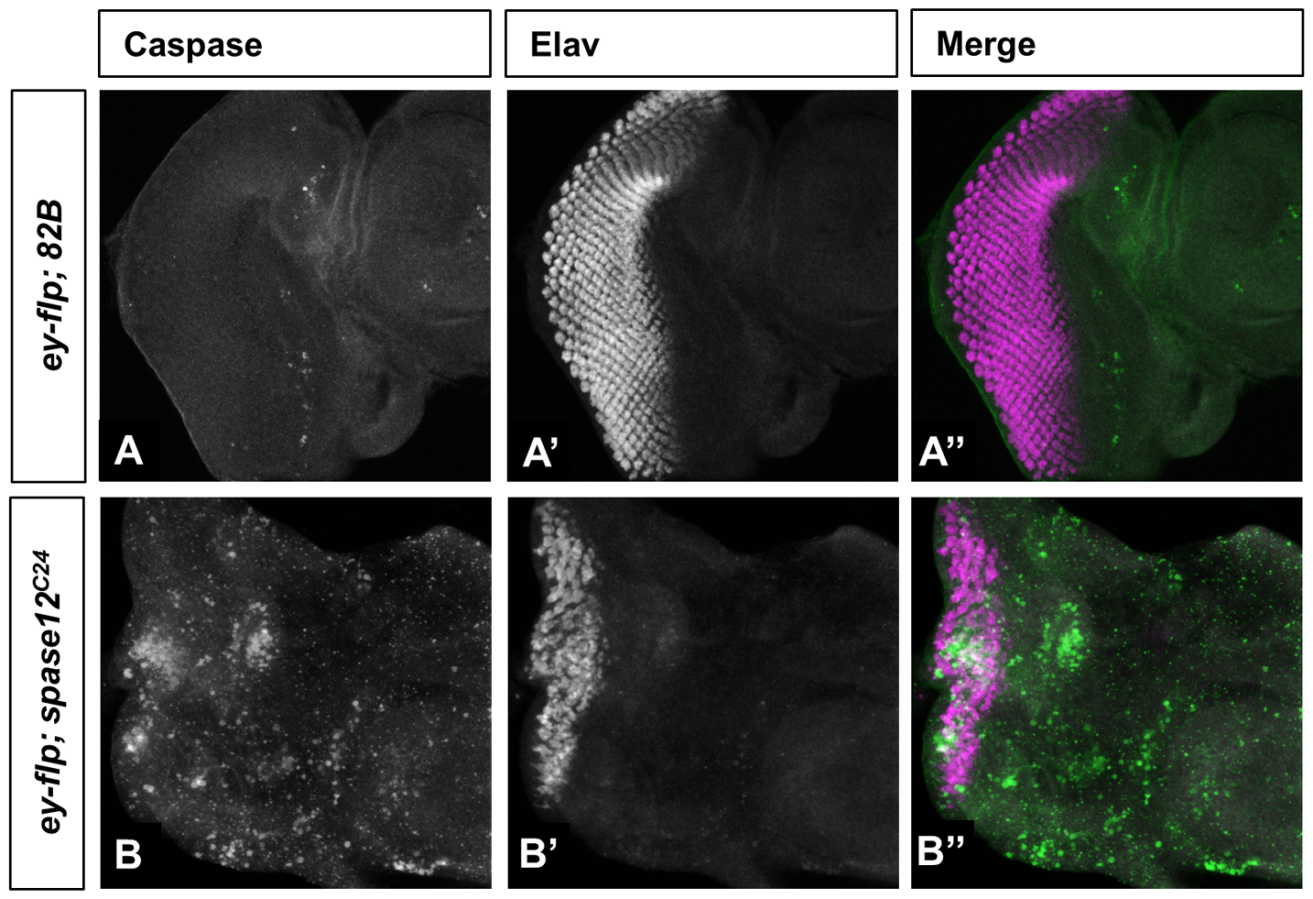
*spase12* LOF leads to increased apoptosis. *yw ey-flp/+; FRT 82B P[w+] cl/FRT* 82B *(ey-flp; 82B)* control (A–A'') has limited Caspase expression and normal Elav expression compared to *yw ey-flp/+; FRT 82B P[w+] cl/FRT 82B spase12^C24^ (ey-flp; spase12^C24^)* (B–B'') mosaic discs in which Caspase is upregulated and Elav expression strongly disrupted.

Notably, melanotic masses have been observed in apoptosis mutants [25] and in LOF *PINK-1 (PTEN-induced protein kinase 1)* mutants with degenerative eye phenotypes [29]. To test whether *spase12* LOF results in increased cell death, *spase12* mosaic third instar discs were stained with Caspase antibody. Increased expression of Caspase was observed in all *ey-flp; spase12^C24^* mutant discs (Figure 5B–B'') that also exhibit severe defects in retinal differentiation as judged by expression of Elav, a pan-neuronal marker. Such disruption and cell death is observed in approximately 10% of larval eye discs examined. Together these data suggest that *spase12* LOF results in disrupted differentiation, increased apoptosis, and melanotic mass formation.

### *spase12* LOF results in cell differentiation errors

The extensive disorganization of the usually stereotypic retina as well as aberrant rhabdomere numbers observed in *spase12* mosaic eyes suggests defects in tissue structure and cell differentiation. Therefore, immunohistochemistry (IHC) was used to examine the structure and appearance of retinal support cells (pigment cells) during pupal development. Using the *hs-flp (M)* method, we generated *spase12^C24^* homozygous mutant tissue in a heterozygous background in which clones are marked by the absence of GFP.

**Figure 6:**
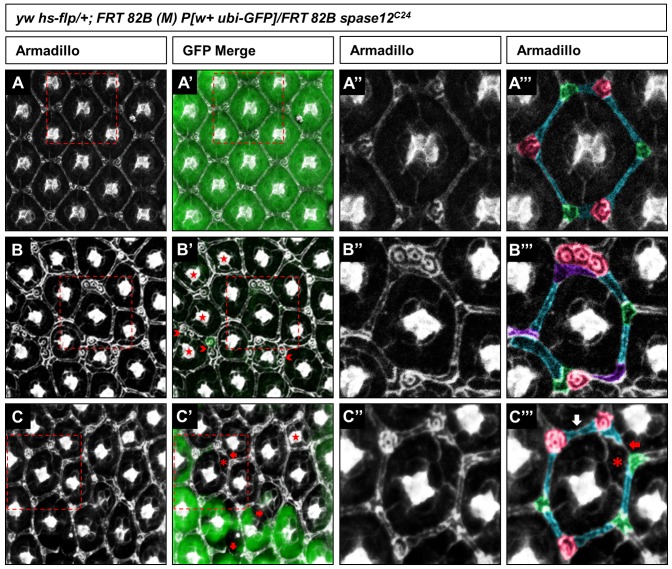
Loss of *spase12* leads to defects in cell differentiation. *yw hs-flp/+; FRT 82B (M) P[w+ ubi-GFP]/FRT 82B spase12^C24^*48 APF eye discs stained with Armadillo (Arm) (A, B, C). GFP negatively marks clones (A', B', C'). Red dashed boxes outline representative ommatidia which are highlighted in A'', B'', C''. Support cells are color-coded according to their identity: interommatidial bristles (magenta), secondary pigment cells (cyan) and tertiary pigment cells (green) (A''', B''', C'''). The center of each ommatidium contains four cone cells, which strongly express Arm, surrounded by two primary pigment cells. *spase12^C24^* mutant tissue (B, B' and C, C') exhibit multiple defects: ommatidia missing one or both primary pigment cells (stars), ectopic IOBs (arrowheads), ectopic primary pigment cell (asterisk), and gaps in the support cell structure that allow contact between primary pigment cells of neighboring ommatidia (red arrows). (B''') A *spase12^C24^* mutant ommatidium is misshapen and has a cluster of three IOBs. Additionally, support cells are not properly placed and the identity of three support cells (purple) cannot be determined by their shape or placement. (C''') A *spase12^C24^* mutant ommatidium fails to maintain the appropriate pattern of cell types at the vertices and possesses an ectopic primary pigment cell (asterisk). A secondary pigment cell (red arrow) does not fully extend to separate one ommatidium from its neighbor and one side of the ommatidium has two secondary pigment cells rather than one (white arrow).

We examined the eye at 48 hours after pupal formation, at which time the pupal eye structure is consistent with the organization of the adult retina. Staining for Armadillo, an adherens junction protein expressed at cell boundaries, marks support cells and cone cells, revealing the precise lattice pattern of the developing eye in control tissue (Figure 6A). Each individual ommatidium forms a hexagonal shape framed by secondary pigment cells intersected with tertiary pigment cells and interommatidial bristles (IOBs) positioned at every other vertex (Figure 6A'''). At the center of each ommatidium is a cluster of four cone cells enclosed by two primary pigment cells. This arrangement is severely disrupted in *spase12^C24^* mutant tissue (Figure 6B''', C'''). In *spase12* clonal tissue, many ommatidia lack the normal hexagonal pattern, instead having only four or five sides or a rounded shape, and some have lost one or both primary pigment cell(s). Additionally, many IOBs and tertiary pigment cells are improperly placed at neighboring vertices, and multiple IOBs may be present at a single vertex. There is also evidence of ommatidial fusions where support cells do not fully surround and enclose each ommatidium (Figure 6B', C'). In the example presented in Figure 6B''', the ommatidium is misshapen, IOBs and tertiary cells are present at only two vertices, three IOBs are located at a single vertex, and the identity of several support cells cannot be determined based on their shape and placement. The ommatidium in Figure 6C''' retains its hexagonal shape, yet IOBs and tertiary cells are not properly positioned in relation to one another, a secondary pigment cell fails to fully extend and enclose the ommatidium resulting in an ommatidial fusion, and an extra primary pigment cell is present. Unlike larval clones where severe defects are observed in only about 10% of discs examined, strong disruption of development in pupal clones is fully penetrant.

**Figure 7:**
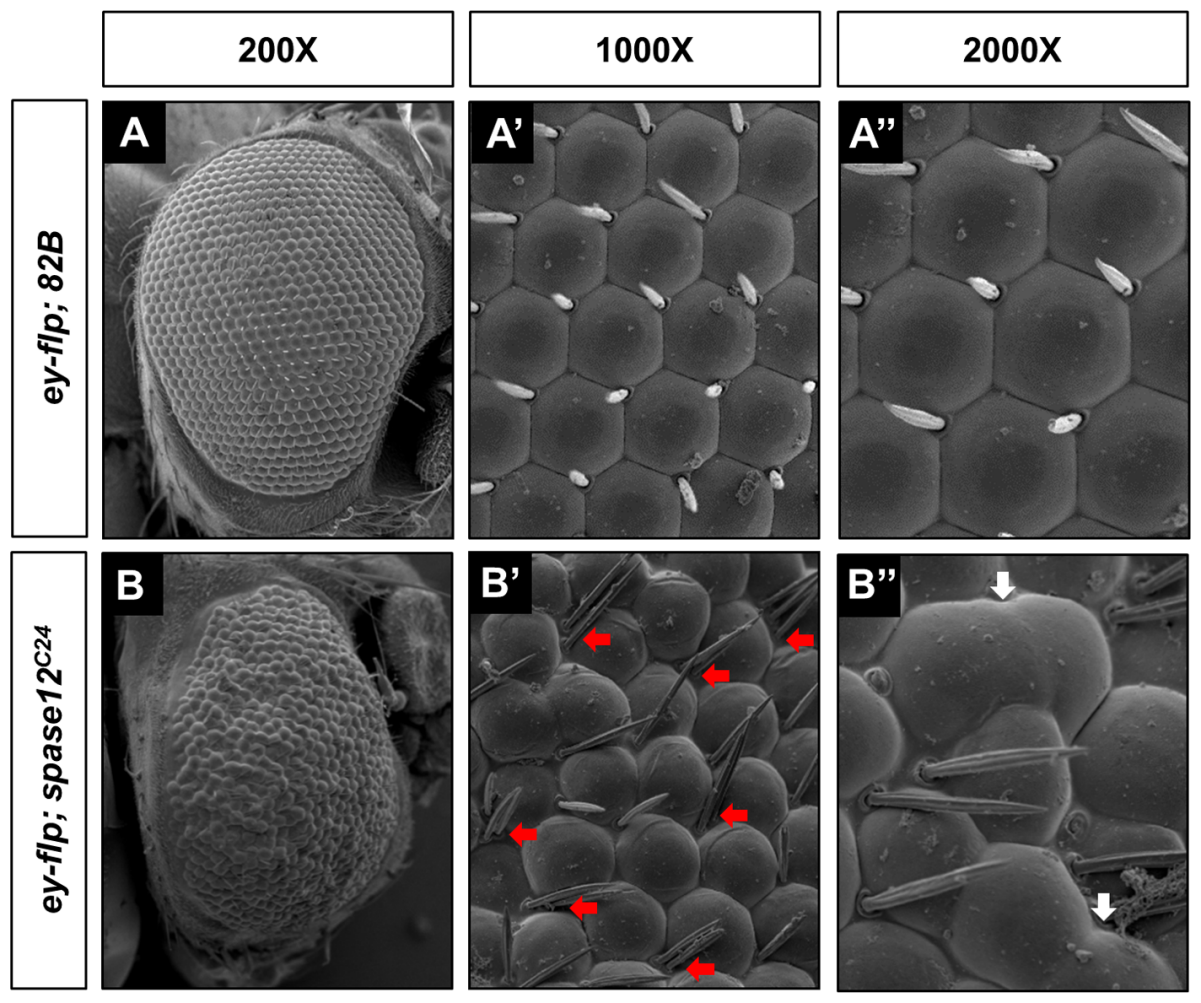
*spase12* LOF results in ectopic IOBs and ommatidial fusions. Scanning electron microscopy (SEM) of (A) *yw ey-flp/+; FRT 82B P[w+] cl/FRT 82B* control and (B) *yw ey-flp/+; FRT 82B P[w+] cl/FRT 82B spase12^C24^*at 200X, (A', B') 1000X, and (A'', B'') 2000X. Adult *ey-flp; spase12^C24^* eyes (B–B'') are disorganized with ectopic interommatidial bristles (red arrows) and ommatidial fusions (white arrows).

Scanning electron microscopy (SEM) was used to image the external surface of the eye to determine whether these defects are maintained in adults and the results are consistent with the phenotypes observed in the pupal eye. The wild-type eye is well organized, with properly placed IOBs (Figure 7A–A''). *ey-flp*; *spase12^C24^* eyes, however, are highly disrupted (Figure 7B–B''). Overall, the eye appears misshapen and the surface lens material of all ommatidia is highly irregular. Consistent with the pupal eye phenotype, bristles are not properly placed at every other vertex, and malformed ommatidia and ommatidial fusions are observed. There are multiple examples of ectopic IOBs in clusters of two and three bristles.

### *spase12* fails to genetically interact with *Notch*

The Notch (N) pathway is a predicted SPC target as N and its ligands, Delta and Serrate, are SP-bearing transmembrane proteins [30]–[32]. Interestingly, *spase12* LOF mosaics exhibit Notch-like phenotypes, in particular, defects in cell differentiation [33]–[35]. However, IHC failed to detect changes in the expression of N or Delta in *spase12* LOF clones. If loss of *spase12* function compromises SPC capacity, it would presumably reduce but not abolish expression of a wide range of proteins – though IHC may be insufficiently sensitive to distinguish changes in expression. To overcome this possibility, we tested whether *spase12* and *N* genetically interact. We generated *N^54l9^/FM7 P[w+ ubi-GFP]; FRT 82B spase12^C24^/TM6B* flies and crossed virgins to *yw ey-flp/Y; FRT 82B P[w+] cl/TM6B* males. Progeny were screened for *yw ey-flp/N^54l9^; FRT 82B cl/FRT 82B spase12^C24^* and *yw ey-flp/FM7 P[w+ ubi-GFP]; FRT 82B cl/FRT 82B spase12^C24^* mosaic flies to determine whether removing a single copy of *N* could exacerbate *spase12* mutant phenotypes. We observed no effect in response to reduced expression of *N*; however, these results do not rule out the possibility of a *spase12-N* interaction (data not shown).

## Discussion

In the current study, we investigated the developmental role of *Drosophila* Spase12, a signal peptidase complex member. Thus far, the SPC has been studied primarily in *S. cerevisiae.* This work represents an effort to expand our understanding of this critical complex in a multicellular model system that is physiologically relevant for the study of human disease. We generated two *spase12* LOF alleles, *spase12^d4^* and *spase12^C24^*, that are recessive and embryonic lethal. Clonal analysis using both of these alleles, and the P-element mutant *spase12^EY10774^*, results in defective development of all tissues tested, including the eye, head, antenna, leg and wing. Further investigation into the retinal defects in *spase12* mosaics reveals increased apoptosis in the developing eye, errors in cell differentiation, disrupted alignment of ommatidia, and melanotic mass formation. From these data we conclude that *spase12* is required for viability and development in *Drosophila*. Although it does not appear to be essential for SPC function, the data show that *spase12* mediates cell differentiation, possibly through regulation of SPC activity on specific substrates or through contributing to SPC efficiency. In addition, this study reveals that loss of *spase12* function causes melanotic mass formation, suggesting that *spase12* LOF may lead to activation of the immune response pathway.

### Deducing the role of *spase12* in the SPC

In yeast, *spc1p* mutants are viable, but accumulate uncleaved pre-proteins in the ER. However, over-expression of *spc1p* rescues lethality of temperature-sensitive *sec11* mutants shifted to the restrictive temperature suggesting that Spc1p contributes to the efficiency of the SPC complex in yeast [5]. Although *Drosophila spase12* LOF is lethal to the animal as a whole, the fact that many *spase12* null mutant cells are viable and can develop and differentiate normally suggests that Spase12, like its yeast homolog Spc1p, is expendable for SPC function. If *spase12* were essential for catalytic function of the SPC, we would expect *spase12* LOF to result in a fully penetrant cell lethal phenotype.

Spase12 may promote SPC cleavage by facilitating the translocation of SPC substrates into the ER. Human SPC12 is predicted to interact with nearly 100 proteins that are directly involved in this process, including more than 70 ribosomal subunits, as well as the translocon, signal sequence binding proteins, and signal recognition particle components [36], [37]. This is consistent with topographical data indicating that mammalian SPC12 is primarily localized to the cytosol [11] where it could interact with ribosomes and translocation machinery on the ER surface. Such interactions may function to stabilize the ribosome-translocon interaction, facilitating the entry of newly translated proteins into the ER.

Loss of *spase12* function causes highly variable phenotypes and does not appear to affect any one specific step in differentiation with full penetrance. For example, in the developing eye we observe both loss and gain of photoreceptors as well as supporting pigment and bristle cells. Furthermore, melanotic masses were observed in only 15% of mosaic animals with varying severity. Cleavage of each of the many SPC substrates may be disproportionately affected from cell to cell and animal to animal resulting in mutable phenotypes and incomplete penetrance. Although strong defects are observed in only 10% of mosaic larval eye discs, disruption of pupal differentiation is fully penetrant, perhaps reflecting a strong maternal contribution of *spase12* transcript that rescues most zygotic null phenotypes into larval stages.

Lethality, however, is a fully penetrant phenotype. The SPC is required for the translocation and subsequent localization of transmembrane and secretory factors, many of which play a role in cell signaling. Even a potentially mild disruption of cell signaling during embryogenesis through loss of *spase12* expression may be an insurmountable obstacle compared to the induction of *spase12* LOF clones at later developmental stages using *ey-flp* or *hs-flp* clonal analysis.

### SPC targets in *Drosophila*

In eukaryotes, all secretory and transmembrane proteins are expected to be translocated to the ER and cleaved by the SPC before they can be properly localized; however, few putative SPC targets have been confirmed in *Drosophila*. *In vitro* experiments provide strong evidence that vitellogenins and Crumbs are cleaved by the SPC. *In vitro* translation of vitellogenins, which are secreted from the fat body, yields preproteins that are significantly larger than endogenously synthesized vitellogenins, suggesting that they are likely to possess an SP that is cleaved by the SPC [38]. In the presence of microsomes derived from either canine pancreas or *Drosophila* embryos, *in vitro* translation of vitellogenins results in polypeptides that are the same size as what is produced *in vivo* [21]. Crumbs, a transmembrane protein with an exceptionally long SP, is co-translationally cleaved in an *in vitro* system to which canine pancreas or *Drosophila* S2 cell-derived microsomes are added [39].

Our investigation in *Drosophila* reveals that pathways populated by SP-bearing proteins may be affected by *spase12* LOF. Loss-of-pigmentation phenotypes in *ey-flp; spase12* mutants suggest that *spase12* may affect the expression of proteins involved in eye pigmentation, such as the pigment cell membrane localized ABC transporter [40]. Additionally, defects in cell differentiation observed in *ey-flp*; *spase12* mutants suggest that cell signaling pathways that function in retinal development, such as Notch, Hedgehog, Dpp, and EGFR [41], may be sensitive to *spase12* expression.

In an effort to identify specific proteins that are disrupted by *spase12* LOF and understand the mechanisms resulting in *spase12* phenotypes, we conducted IHC in third instar and pupal *spase12* mosaic eye discs. Expression of Crumbs, a known SPC substrate, was unaltered in *spase12* mosaic tissue. Additionally, IHC failed to detect any changes in the expression of DE-Cadherin, Fasciclin 2, and Notch pathway members Notch and Delta – all of which are SP-bearing, transmembrane proteins. These results, however, do not rule out the possibility that the expression and function of these potential targets are impacted by *spase12* LOF. If Spase12 promotes the overall activity of the SPC but is not absolutely required for SPC function, there may not be a sufficient reduction in the expression or localization of any one protein to be detected with IHC. Conversely, Spase12 may not be necessary for SPC cleavage and subsequent localization of the putative SPC substrates tested. Because *spase12* LOF in the entire animal is lethal, S2 cells may present a viable alternative to identify SPC substrates that require *spase12* expression for localization and function. Partial RNAi knockdown of *spase12* in S2 cells, coupled with quantitative approaches to assay the expression levels of putative SPC targets may aid the identification of proteins affected by *spase12* LOF.

### Melanotic masses in *spase12* mutants

*spase12^C24^* mosaic flies are susceptible to melanotic mass formation. Mutated genes that result in melanization can be divided into two groups. Class I genes are not involved with the immune pathway, but may induce an immune reaction when altered in response to abnormal or degenerating tissue that is recognized and attacked by the immune system. Class II genes include those known to function in immune response pathways such as *Toll* and *JAK/STAT* [42], [43].

Although *spase12* mosaic animals develop melanotic masses, it is unclear whether Spase12 functions directly in immunity pathways. However, melanotic mass formation is executed by hemocytes which circulate freely in the hemolymph and throughout the organism. Therefore, mutations that promote melanization are likely to result in a systemic phenotype [25]. In contrast, melanotic mass formation in *spase12* mosaic flies is restricted to the mutant tissue, suggesting that *spase12* may be a Class I gene.

Several studies have suggested that melanotic masses correlate with both necrotic and apoptotic cell death. In *necrotic (nec)* mutants, necrosis was shown to correspond with melanotic mass formation [44]. *PINK-1 (PTEN-induced protein kinase 1)* LOF in the eye results in photoreceptor degeneration, melanizations, and necrosis [29]. Mutations in apoptosis genes *dronc (Nedd2-like caspase)*, *dcp-1 (Decapping protein 1)* and *ark (Apaf-1-related-killer)* also result in melanotic mass formation [25]. We observe an increase in apoptotic cell death in developing *spase12* mosaic eyes, and melanotic masses, degeneration, and evidence of cell death in adult eyes suggesting a correlation between cell death and melanization in *spase12* mutant tissue.

It is also interesting to note that *Drosophila* homologs of five of the proteins that human SPC12 is predicted to interact with are linked with melanotic mass formation and each has a role in protein synthesis and translocation into the ER [36], [37], [45]. These include Gtb-bp and Srp54k, which target SP-bearing proteins to the ER, and ribosomal subunits RpL26, RpL6, and RpS5b [45]. Further investigation into the link between *spase12* LOF, melanotic mass formation, and cell death may shed light on the functional role of Spase12 in the SPC.

In this report we have shown that *Drosophila* Spase12 is required for viability, development, and cell differentiation. Furthermore, *spase12* LOF lethal results in increased incidence of cell death in the developing eye, as well as retinal degeneration and melanotic mass formation in adults. This work demonstrates that, in *Drosophila*, *spase12* is essential to development in higher eukaryotes, and suggests that future studies investigating the function of Spase12 may enhance our understanding of the intricacies of protein translocation regulation.

## Methods

### Drosophila stocks

Flies were cultured at 25°C on standard media. The following stocks were used: *ubi-gal4*, *spase12^d4^ P[w+]*, *spase12^C24^*, *spase12^rev^*, *spase12^EY10774^ P[w+]* [46], *yw ey-flp/+*; *FRT 82B P[w+] cl/TM6B*, *yw hs-flp/+*; *FRT 82B (M) P[w+ ubi-GFP]/TM6B*, *yw ubx-flp/+*; *FRT 82B (M) P[w+ ubi-GFP]/FRT 82B/TM6B*, *UAS-spase12*, *spase12 GR* and *N^54l9^/FM7 P[w+ ubi-GFP]*. *spase12^d4^ P[w+]* was generated using FRT-bearing transposons *e01743* and *d06279* (Exelixis) as described [47]. *spase12^rev^*and *spase12^C24^* were generated via precise and imprecise excision, respectively, of *spase12^EY10774^ P[w+]* using □ *2–3* transposase following standard methods.

### Constructs

*UAS*-*spase12* was generated by inserting *spase12* cDNA (*RE02772*, DGRC) into *pUAST-attB* [48]. *spase12 GR* was generated by recombineering a 29 kb fragment of *BACR28B07* into *p[ACMAN*] [49]. *p[ACMAN]* was a gift from Hugo Bellen (Jan and Dan Duncan Neurological Research Institute, Houston, TX, USA). Transgenics were generated by injection into *VK1* (*UAS-spase12*) and *P2* (*spase12 GR*) [49].

### Clonal analysis

*spase12^d4^ P[w+], spase12^EY10774^ P[w+] *and *spase12^C24^* were recombined onto *FRT82B*. Clonal analysis was conducted using *yw ey-flp/+; FRT 82B P[w+] cl/TM6B, yw hs-flp/+; FRT 82B (M) P[w+ ubi-GFP]/TM6B, and yw ubx-flp/+; FRT 82B (M) P[w+ ubi-GFP]/FRT 82B/TM6B* stocks. *hs-flpclones* in pupal eye discs were generated via a 1 hour heat shock at 37°C to induce *hs-flp expression* at 48 hours after egg lay. Pupal discs were dissected 48 hours after pupation.

### Immunohistochemistry and confocal microscopy

Antibody staining of third instar and pupal eye discs was performed as described [50]. The following primary antibodies were used: rabbit anti-GFP (1∶1000, Molecular Probes), mouse anti-GFP (1∶1000, Molecular Probes), rabbit anti-Caspase (Cell Signaling), mouse anti-Arm (1∶500, DSHB) [51], and rat anti-Elav (1∶500, DSHB) [52]. The following secondary antibodies were used: Alexa goat anti-rabbit (1∶500, Molecular Probes), Alexa goat anti-mouse (1∶500, Molecular Probes), CY3 goat anti-mouse (1∶500, Jackson ImmunoResearch), CY5 goat anti-rabbit (1∶500, Jackson ImmunoResearch), and CY3 goat anti-rat (1∶500, Jackson ImmunoResearch). Images were captured using a Zeiss LSM 510 confocal microscope (Zeiss, Jena, Germany) and processed with ImageJ (NIH, Bethesda, MD, USA) and Adobe Photoshop software (Adobe Systems Incorporated, San Jose, CA, USA).

### Thin plastic sections and light microscopy

Thin plastic, tangential sections of the adult retina were performed as described [53]. Images of external *Drosophila* morphology and thin plastic sections were captured using a Zeiss Axioplan 2 microscope, Zeiss AxioCam digital camera and AxioVision software and processed with ImageJ (NIH, Bethesda, MD, USA) and Adobe Photoshop software (Adobe Systems Incorporated, San Jose, CA, USA).

### Electron microscopy

Flies were prepared and fixed in HMDS as described [54]. The samples were then coated under vacuum using a Balzer MED 010 evaporator (Technotrade International, Manchester, NH) with platinum alloy for a thickness of 25 nm, then immediately flash carbon coated under vacuum. The samples were transferred to a desiccator for examination at a later date. Samples were examined in a JSM-5910 scanning electron microscope (JEOL, USA, Inc., Peabody, MA) at an accelerating voltage of 5 kV.
